# Assessment of Non-Consumptive Predation Risk of *Coccinella septempunctata* (Coleoptera: Coccinellidae) on the Population Growth of *Sitobion miscanthi* (Hemiptera: Aphididae)

**DOI:** 10.3390/insects13060524

**Published:** 2022-06-06

**Authors:** Liping Wang, Remzi Atlihan, Ruirui Chai, Yao Dong, Chen Luo, Zuqing Hu

**Affiliations:** 1 State Key Laboratory of Crop Stress Biology in Arid Areas, College of Plant Protection, Northwest A&F University, Yangling 712100, China; wanglip111@163.com (L.W.); 2017010299@nwafu.edu.cn (R.C.); dyinthebox@163.com (Y.D.); lczhibao@163.com (C.L.); 2 Department of Plant Protection, Faculty of Agriculture, Van Yüzüncü Yıl University, 65080 Van, Turkey; ratlihan@yyu.edu.tr

**Keywords:** predation risk effects, seven-spot ladybird beetle, grain aphid, the age-stage, two-sex life table

## Abstract

**Simple Summary:**

Changes in prey biology driven by predation threats that do not involve direct consumption are referred to as non-consumptive effects (NCEs). In general, NCEs are considered common and can affect herbivores sometimes stronger than the direct consumptive effects. However, how the NCEs of predators affect the development, survival, fecundity, and population growth of prey has not been well documented, which is the primary consideration for the compatibility of prey with its natural enemies in agricultural ecosystems. We examined the NCEs of the predator *Coccinella septempunctata* on the life-history traits and population growth of *Sitobion miscanthi* via caged predator (i.e., *S. miscanthi* co-existed with caged *C. septempunctata*) and caged prey (i.e., *C. septempunctata* co-existed with caged *S. miscanthi*) treatments by employing the age-stage, two-sex life table. The findings indicate that *S. miscanthi* could respond to the predation risk of caged predators by either accelerating the developmental rate or reducing the net reproductive rate, while *S. miscanthi* might reduce their fitness in response to the predation risk of caged prey. Furthermore, *S. miscanthi* might also increase the number of winged morphs under both of the above treatments. The results have practical ramifications on managing this economically important pest on wheat production with reduced insecticide applications.

**Abstract:**

How the non-consumptive effects (NCEs) of predators influence the development, survival, fecundity, and population growth of prey has not been well documented, which is the primary consideration for the compatibility of prey with its natural enemies in agricultural ecosystems. We herein employed the age-stage, two-sex life table to examine the NCEs of the predator *Coccinella septempunctata* on the life-history traits and population growth of prey *Sitobion miscanthi* via caged predator (prey co-existing with caged predator) and caged prey (predator co-existing with caged prey) treatments with daily different exposure times (i.e., 0 h (control), 12 h, and 24 h). The results indicated that the predation risk of a caged predator could reduce the first nymphal duration and net reproductive rate (*R*_0_) of *S. miscanthi* at 12 h, and the first nymphal duration, preadult duration, and mean generation time (*T*) at 24 h. However, the predation risk of the caged prey resulted in the prolongation of the pre-adult development time and total pre-reproductive period (TPRP) as well as lowered the intrinsic rate of increase (*r*), finite rate of increase (𝜆), *R*_0_, life expectancy, and reproductive value of *S. miscanthi* after both 12 h and 24 h. Furthermore, the predation risk of both the caged predator and caged prey could increase the percent of winged morph at 24 h. These findings indicate that *S. miscanthi* could respond to the predation risk of the caged predator by either accelerating the developmental rate or reducing the net reproductive rate, while *S. miscanthi* might reduce their fitness in response to the predation risk of caged prey. Furthermore, *S. miscanthi* might also alter to winged morphs for dispersal under both of the above treatments. The findings obtained have practical ramifications for managing this economically important pest in wheat production with reduced insecticide applications.

## 1. Introduction

The predator–prey interaction is the most common basic relationship in insect ecology, which is of great significance for the development of ecological communities of farmland and pest management [1,2]. Predators can alter the prey abundance via consumption or threat. In turn, the prey can also detect and utilize a variety of cues to avoid encounters with their predators. The common method used by prey is to alter their fitness [3,4] and to migrate (for instance, prey aphids release alarm pheromones that allow other aphids to escape from predators [5,6]). Biological changes in prey driven by predation threat that does not involve direct consumption are termed non-consumptive effects (NCEs), also referred to as risk effects. NCEs can ultimately impact the prey abundance by altering the prey phenotype (e.g., behavior, morphology, or physiology) [7]. NCEs increase the complexity of the prey and predator interactions, as there can be either positive or negative effects of the predator on the prey. This knowledge can add to our understanding of how biological control agents impact pests [8]. In general, NCEs are considered common and can affect herbivores as strongly as and sometimes stronger than the direct consumptive effects [9]. Biological control in agricultural ecosystems seeks to augment insect natural enemies to suppress pest populations; therefore, a further understanding of how NECs alter the predator–prey dynamics is of particular importance [10].

To date, the related studies of NCEs of arthropod predators on prey have mainly focused on behavioral and physiological changes including changes in habitat use [11,12], feeding behavior [13], oviposition [14,15], colonization or dispersal [16], life history adjustment [17], metabolic rate [18], and the expression level of stress proteins (e.g., heat shock proteins [19]). For example, under the predator convergent lady beetle (*Hippodamia convergens* (Guerin) (Coleoptera: Coccinellidae)) risk, the prey potato aphids (*Macrosiphum euphorbiae* Thomas (Hemiptera: Aphididae)) reduced the population density [20]. Furthermore, the threat of the predator *Coccinella septempunctata* L. (Coleoptera: Coccinellidae) also increased the formation of alates of the prey *Rhopalosiphum padi* (L.) (Hemiptera: Aphididae) [21]. Generally, prey can adapt by modifying their phenotypes to avoid encounters with predators (i.e., lower predation risk). In the biological control arena, if only the consumptive effect of a predator on a target pest population is considered, large numbers of predator individuals are required; however, if the NCEs have adequate impacts on pest populations, relatively small numbers of predators are required to keep the pest numbers below economic injury levels [22]. However, how the NCEs of predators affect the development, survival, fecundity, and population growth of prey, which is the primary consideration for the compatibility of natural enemy insects, is not understood comprehensively.

The grain aphid, *Sitobion miscanthi* (Takahashi) (Hemiptera: Aphididae), (widely misreported as *Sitobion avenae* (Fabricius) (Hemiptera: Aphididae) in China [23]), is an important pest in cereal crops. The piercing-sucking mouthparts of the aphid remove the wheat phloem sap and transmit the plant virus, which seriously affects the wheat growth and reduces wheat production [24,25,26,27]. *S. miscanthi* has several types of natural enemies, which is an important factor for its natural control of aphid populations. The seven-spotted lady beetle, *C*. *septempunctata*, is one of the important predatory natural enemies of *S. miscanthi*, and its population density is high in wheat fields [28]. In order to accurately assess the effectiveness of a natural enemy and its success as a biological control agent, the NCEs and consumption effects need to be evaluated simultaneously. Many studies have shown that the lady beetle consumption effects on *S. miscanthi* might play a significant role in the ecological regulation of pest populations in the wheat field ecosystem [29]. However, the NCEs of *C. septempunctata* on *S. miscanthi* have not been previously reported. 

Life tables are composed of comprehensive datasets regarding the survival, development, and fecundity of a population [30,31]. As a powerful tool for data analysis, insect life tables have been extensively used in fundamental research in insect population ecology and integrated pest management tactics [32,33,34]. In order to determine the variability of the prey population dynamics under the NCEs, we conducted life table studies of *S. miscanthi* under predatory risk by the predator *C. septempunctata*. These results provide critical baseline information for predator–prey interactions and pest management programs in general.

## 2. Materials and Methods

### 2.1. Collection and Maintenance of Insects 

The grain aphid *S. miscanthi* was collected as a single parthenogenetic female from a wheat field near Yangling, Shaanxi Province, China (34.28° N, 108.22° E). Wheat seedlings (*Triticum aestivum* cv. ‘Aikang58’) were grown as the host plant in the bioclimatic chamber at 20 ± 1 °C, 75 ± 5% RH, and a 16L:8D h photoperiod. The wheat plants were enclosed within 30 cm × 30 cm × 30 cm cages. *S. miscanthi* individuals were reared for more than thirty generations under predator-free conditions prior to their use in the current experiment. 

The predator *C. septempunctata* individuals were collected from wheat fields at the same location as the aphid clone described above and maintained in growth chambers under the same environmental conditions for more than eight generations prior to the onset of the experiments and supplied with fresh *S. miscanthi* at all growth stages. The adults of *C. septempunctata* within 24 h after emergence were isolated, and the 3-d old unmated female adults (starved for 24 h in advance) were used for the experiments [35,36].

### 2.2. Experimental Design and Life Table Study

**Figure 1:**
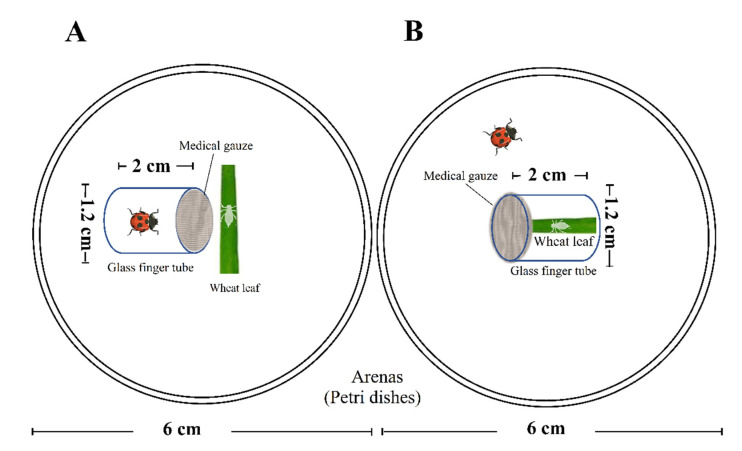
The experimental design for the caged predator model (**A**) and caged prey model (**B**). n = 30.

All experiments were conducted in the chamber conditions as shown above. Two types of predation risk treatments were employed, named, “caged predator” and “caged prey” (Figure 1); three different exposure durations per day were used in each treatment. The details are as follows:

“Caged predator” treatments: A single nymph (<12 h old) of *S. miscanthi* produced by a newly developed parthenogenic wingless adult aphid was transferred to a Petri dish (Ø 6 cm and 2.1 cm height) containing fresh wheat leaves (3 cm length). After 12 h of adaptation, a glass finger tube (Ø 1.2 cm and 2 cm height; sealed with medical gauze) with one randomly chosen newly emerged *C. septempunctata* female adult inside was transferred into the Petri dish with the aphid (Figure 1A). In the experiment, three treatments of exposed times [i.e., 0 h (control, transferred with empty glass finger tube), 12 h (half-day), and 24 h (whole day)] were employed. Every day, after being exposed for different times, the *C. septempunctata* was removed from the glass finger tube, and the tube was kept empty. An initial number of 30 nymphal aphids were used for each treatment. Wheat leaves were replaced with fresh ones every two days.

“Caged prey” treatments: the method was similar to the “caged predator” treatments, however, the aphid nymph was placed in the glass finger tube containing fresh wheat leaves (2 cm length), and the predator was in the Petri dish (Figure 1B). Every day, after being exposed for different times, the *C. septempunctata* was removed from the Petri dish.

Every 24 h, the development and survival of the immature stages were recorded for each individual. The adult pre-reproductive period (APRP), total pre-reproductive period (TPRP), number of nymphs produced, number of reproductive days, and adult longevity were all calculated after adult emergence. Newborn nymphs were removed from Petri dishes after their numbers were recorded during observations. The type of adult morph (winged or wingless adult) was also recorded.

### 2.3. Life Table Analysis

The life-history traits data, in other words, the development time, survival, longevity, adult pre-reproductive period (APRP; the duration from adult emergence to the first reproduction), total pre-reproductive period (TPRP; the duration from birth to the first reproduction), and fecundity of *S. miscanthi* were analyzed according to the age-stage, two-sex life table theory using the computer program Twosex-MSchart [37]. The population parameters estimated in the life table, their definitions, and equations used in the calculations are listed in Table 1. 

The variances and standard errors of the biological and population parameters were estimated using the bootstrap resampling method with 100,000 iterations [41,45,46]. The differences between treatments were compared by using the paired bootstrap test based on the confidence interval of differences (5% significance level) [47,48,49]. The population growth of *S. miscanthi* for a 60 d duration was predicted using the computer program TIMING-MSChart [32,50,51]. The initial population consisted of 10 neonate nymphs.

The reproductive period considering the duration from the first to the last reproduction has been widely used in demographic studies. However, the actual number of days that an insect or mite has produced offspring is not equivalent to this period (i.e., a long reproductive period may not necessarily represent a high reproduction). Therefore, in this study, the parameter of reproductive days (*R_d_*), which reflects the actual number of days that a female has produced offspring, was used instead of the reproductive period. The reproductive days was calculated according to Chen et al. [52] as:

$$R_{d}=\frac{\sum_{x=1}^{N_{fr}}D_{x}}{N_{fr}}$$

where *R_d_* is the reproductive days; *N_fr_* is the reproductive females (i.e., those females that actually produce live nymphs); *D_x_* is the number of reproductive days of a single adult female.

## 3. Results

### 3.1. Nymphal Development

The immature developmental durations of *S. miscanthi* exposed to different predation risks are presented in Table 2. The results indicate that exposure to different predation risks affected the developmental time of the pest. Under the predation risk of the caged predator, the developmental time of the first nymphal stage aphid after 12 h and 24 h exposure time treatments was shorter than that obtained in the control treatment (*p =* 0.0010, *p =* 0.0003), leading to the shorter total preadult development time. The shortest total preadult developmental time was obtained with 24 h exposure time treatment (*p =* 0.0350), followed by 12 h exposure time and control treatments; however, there was no difference between the control and 12 h exposure time treatment, nor between the 12 h and 24 h exposure time treatments. In contrast to the caged predator treatment, the total preadult development time of the pest was prolonged in the caged prey treatment compared to the control treatment (*p =* 0.0017, *p =* 0.0006) (Table 2). This was due to the prolonged developmental time of the first and third nymphal stage for the 12 h and 24 h exposure time treatments. 

The duration of the first nymphal stage in the caged prey trial was shorter at 0 h (*p =* 0.0019) and longer at exposure times of 12 and 24 h (*p =* 0.0059, *p =* 0.0252) compared to the caged predator trial. There was no difference in the duration of the second and third nymphal stages between the caged prey and caged predator trials at all exposure times. However, the duration of the fourth nymphal stage at 24 h (*p =* 0.041) and total preadult duration at exposure times of 12 and 24 h (*p =* 0.0055, *p =* 0.0043) were significantly shorter in the caged predator trials (Table 2).

The effects of exposure duration to different predation risks on the survival rate of the pest are shown in Table 2. Exposure to the predation risk of the caged predator for different durations (0, 12, or 24 h) did not change the pest’s survival rate. However, under the risk of the caged prey, the survival rate of the pest changed based on the exposure duration; the survival rate was the highest in the control treatment (0 h), and the lowest in the exposure of the 12 h treatment. However, the difference between the results obtained at 0 h and 24 h, and 12 h and 24 h treatments was insignificant (*p* > 0.05). Thus, there was no difference in the survival rate of the pest between the caged prey and caged predator trials at all exposure times.

### 3.2. Adult Longevity, Reproduction, and the Percent of Winged Morphs

Table 3 displays the APRP (adult pre-reproductive period), TPRP (total pre-reproductive period), adult longevity, total longevity, reproductive days, fecundity, and the percent of winged morphs of *S. miscanthi* exposed to different predation risks. Under the predation risk of the caged predator, exposure for 24 h resulted in a higher percentage of winged morphs than the control treatment (*p* < 0.001), and there was no difference between the cohorts exposed to different durations (i.e., 0, 12, and 24 h) in terms of the other biological parameters above-mentioned (Table 3). However, exposure for 12 h and 24 h to caged prey treatment resulted in a longer TPRP duration than the control treatment (*p =* 0.0095, *p =* 0.0135), and exposure for 24 h showed a higher percentage of winged morphs than the control treatment (*p* < 0.001). Exposure to caged prey treatment did not change the other parameters, as shown in Table 3.

Furthermore, exposure to different predation risks did not affect the APRP, total longevity, and the percentage of winged morphs of the pest. The TPRP values obtained for the aphid cohorts exposed for 12 h and 24 h to the caged predator treatment were lower than those obtained in the caged prey treatment (*p =* 0.0296, *p =* 0.0044); however, the exposure to the caged predator treatment for 24 h caused a longer adult longevity than the caged prey treatment at the same exposure duration (*p =* 0.0131). Exposure to caged prey for a duration of 0 h (control) and 24 h resulted in a lower number of reproductive days from the caged predator treatment. The fecundity values obtained under the caged prey treatment at 0, 12, and 24 h were significantly lower than those obtained under the caged predator treatment (*p =* 0.001, *p =* 0.0058, *p* < 0.001) at the same duration of exposure (Table 3).

### 3.3. Life Table and Population Parameters

**Figure 2:**
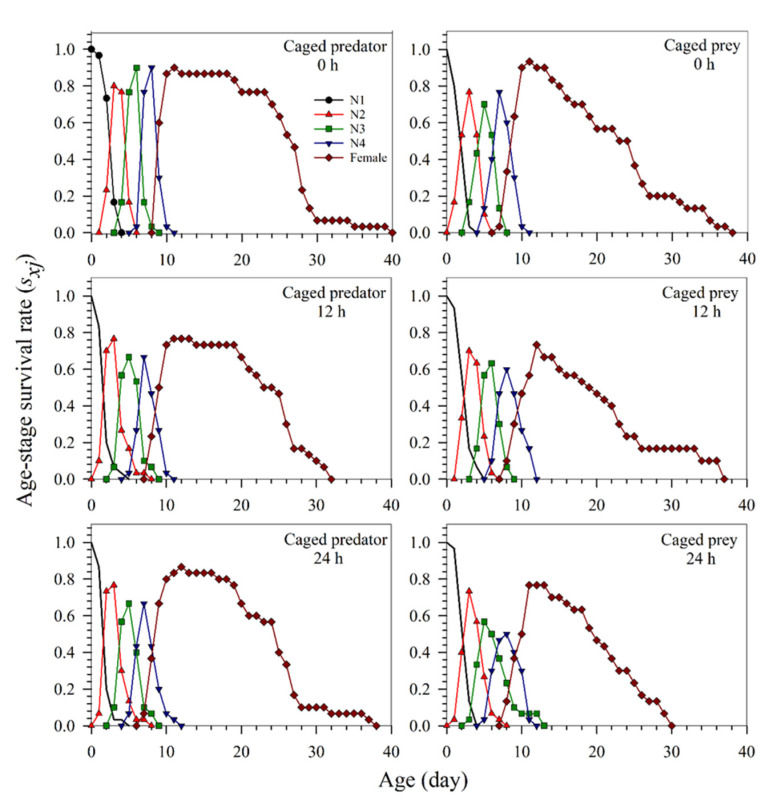
The age-stage specific survival rates (*s_xj_*) of *Sitobion miscanthi* under predator *Coccinella septempunctata* risk at different exposure times (0, 12, and 24 h) with either the caged predator or caged prey treatments. N: Nymphal instar of *S. miscanthi.*

The age-stage specific survival rate (*s_xj_*) of *S. miscanthi* shows the probability that a newly born nymph will survive to age x and stage j after exposure to the caged predator and caged prey systems for 0, 12, and 24 h, respectively (Figure 2). The results indicate that the probability of neonate nymphs surviving to the adult stage was the highest in the control treatment (0 h) and the lowest in the 12 h treatment in both the caged predator and caged prey systems. When exposed to 0, 12, and 24 h durations, the probability of the neonate nymphs surviving to the adult stage was 90, 77, and 87% in the caged predator system, and 93, 73, and 77% in the caged prey system, respectively. The results in the caged prey system at the 24 h exposure were different from the preadult survival rate of 80% (Table 2). The mentioned inconsistency was due to the fact that certain adults emerged earlier and died before other adults emerged. The results obtained in both systems at the same exposure duration were similar. In Figure 2, the variable rate of development among individuals is also graphically illustrated with overlap between stages. 

**Figure 3:**
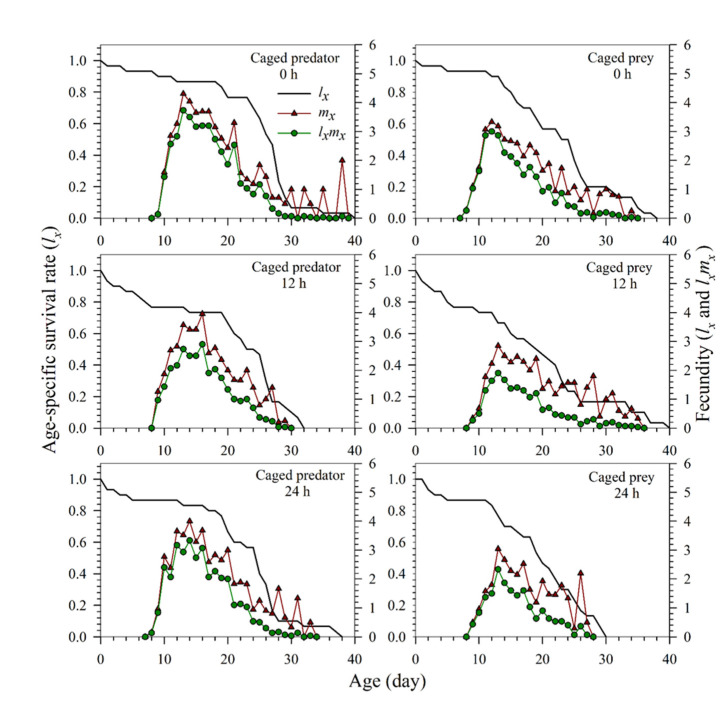
The age-specific survival rates (*l_x_*), age-specific fecundity (*m_x_*), and net maternity (*l_x_m_x_*) of *Sitobion miscanthi* reared under predator *Coccinella septempunctata* risk at different exposure times (0, 12, and 24 h) with either the caged predator or caged prey treatments.

The age-specific survival rate (*l_x_*), age-specific fecundity (*m_x_*), and age-specific maternity (*l_x_m_x_*) of *S. miscanthi* cohorts treated with different predation risks are shown in Figure 3. The curve of *l_x_* was a summation of the survival curves at different stages at age *x*. The highest *m_x_* and *l_x_m_x_* peaks occurred in the control treatments under both predation risk of the caged predator and caged prey. Under the predation risk of the caged predator, the highest peaks of *m_x_* occurred on day 13 with 4.3 offspring in the control (0 h), day 16 with 3.95 offspring at 12 h, and day 12 with 3.65 offspring at 24 h. Under the predation risk of caged prey, the highest peaks of *m_x_* occurred on day 12 with 3.33 offspring in the control (0 h), day 16 with 2.47 offspring at 12 h, and day 13 with 3.04 offspring at 24 h. The age-specific maternity (*l_x_m_x_*) curves showed a similar tendency. 

Exposure to the predation risk of the caged predator did not affect the *r* and λ; there was no difference between the values obtained in the control (0 h) treatment and the two different exposure treatments (12 and 24 h). However, the *R*_0_ value at the exposure of 12 h and *T* value at the exposure of 24 h was significantly lower than that in the control treatment (*p =* 0.0494; *p =* 0.0036). Exposure to the predation risk of caged prey significantly affected the population parameters of the pest. Compared to the control treatment, the higher r, λ, and *R*_0_ values at the 12 h and 24 h exposure times (*r*: *p =* 0.0005, *p =* 0.0016; λ: *p =* 0.0011, *p =* 0.0052; *R*_0_: *p =* 0.0231, *p =* 0.0124) and a longer *T* value at the 12 h exposure time (*p =* 0.0202) were obtained (Table 4). 

The life table results indicate that exposure to the predation risk of the caged prey affected the pest more than exposure to the predation risk of the caged predator. The *r*, λ, and *R*_0_ values, which reflect the combined effects of biological parameters such as developmental time, survival, and reproduction at 12 and 24 h exposure durations were significantly lower under the caged prey treatment from the values in the caged predator treatment (*r*: *p =* 0.0102, *p =* 0.0012; λ: *p =* 0.0097, *p =* 0.001). Moreover, in the control treatment, the *R*_0_ value obtained in the caged prey treatment was lower than that obtained in the caged predator treatment (Table 4). However, the *T* value under the caged prey treatment at 0 h (control) was significantly shorter than that at the caged predator treatment (*p =* 0.0014) (Table 4).

**Figure 4:**
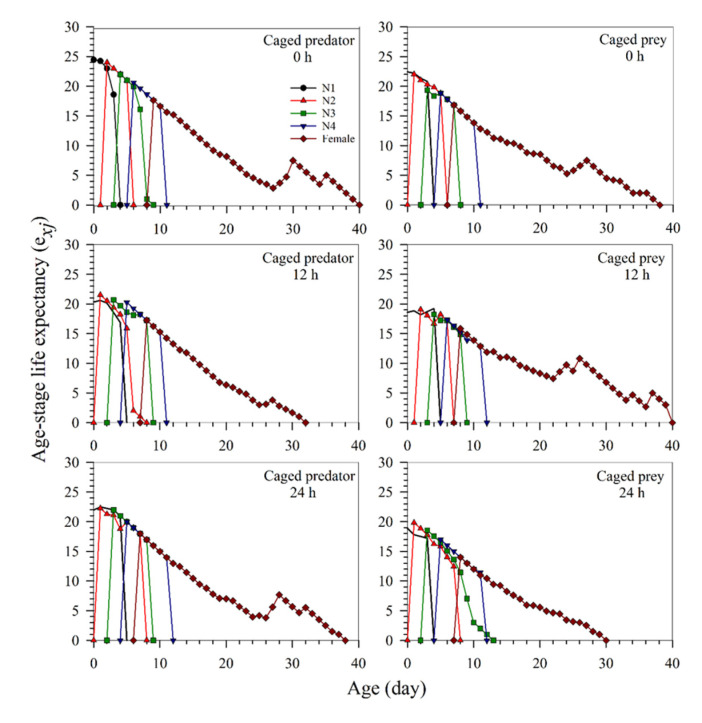
The age-stage specific life expectancy (*e_xj_*) of *Sitobion miscanthi* reared under predator *Coccinella septempunctata* risk at different exposure times (0, 12, and 24 h) with either the caged predator or caged prey treatments. N: Nymphal instar of *S. miscanthi.*

The life expectancy (*e_xj_*) of *S. miscanthi* treated with different predation risks is shown in Figure 4. The life expectancy of the pest at age zero (i.e., *e*_01_) at the 0, 12, and 24 h exposure times was longer at the caged predator risk than under the caged prey risk. The *e*_01_ values of the pest at the 0, 12, and 24 h exposure times were determined as 24.43, 20.33, and 21.97 d in the caged predator treatment, and 22.43, 18.60, 18.87 d, respectively, in the caged prey treatment. The life expectancy of a neonate nymph was the same as the mean longevity of all individuals used in the life table study. 

**Figure 5:**
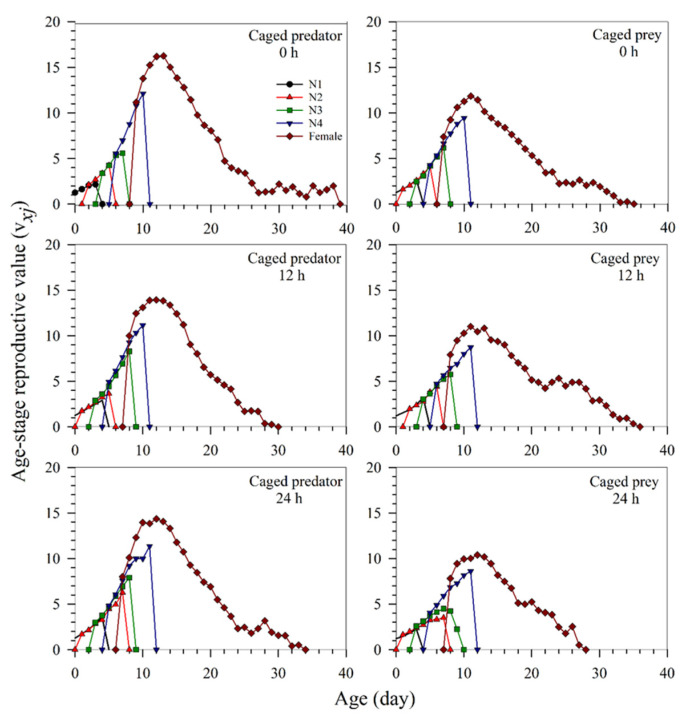
The age-stage specific reproductive value (*v_xj_*) of *Sitobion miscanthi* reared under predator *Coccinella septempunctata* risk at different exposure times (0, 12, and 24 h) with either the caged predator or caged prey treatments. N: Nymphal instar of *S. miscanthi.*

The reproductive value of a neonate nymph (i.e., *v*_01_, Figure 5) was the same as the finite rate (λ), as shown in Table 4. The major peak occurrence time of the reproductive values of females under the predation risk of the caged predator and caged prey were similar; however, the higher peak values were obtained with the caged predator treatment. The highest peak values of the pest cohorts at the exposure times of 0, 12, and 24 h were 16.25 (day 13), 13.93 (day 12), and 14.35 (day 12) in the caged predator treatment, respectively; and 11.85 (day 11), 11.02 (day 11), and 10.39 (day 12) in the caged prey treatment, respectively (Figure 5). The higher peaks in the caged predator treatment indicate that the aphid population will increase faster in this treatment than that of the caged prey treatment, irrespective of the exposure time durations (0, 12, or 24 h) (Figure 5).

### 3.4. Population Projection of S. miscanthi

**Figure 6:**
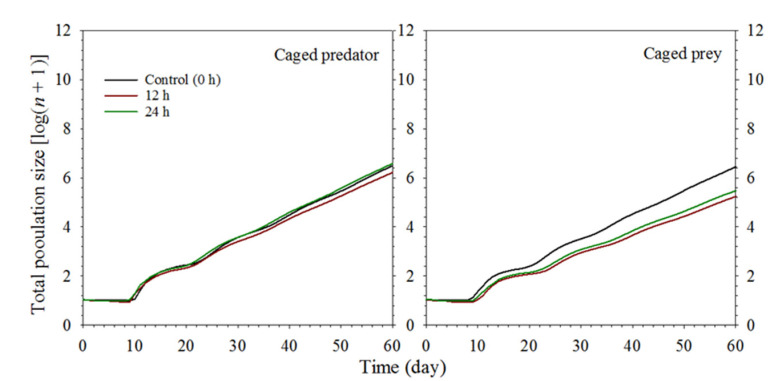
Population projections for *Sitobion miscanthi* reared under predator *Coccinella septempunctata* risk at different exposure times (0, 12, and 24 h) with either the caged predator or caged prey treatments.

The projected total population size results of *S. miscanthi* under two different predation risks are shown in Figure 6. The population projection results in the logarithmic scale indicated that the lowest population growth rate was obtained at the exposure of 12 h under the predation risk of the caged predator. The population growth rate at the exposure of 24 h showed a similar trend as the control (0 h exposure) treatment. However, under the predation risk of the caged prey, the population growth rate at the control treatment was considerably higher than those exposed to the 12 and 24 h treatments. As with the caged predator treatment, the lowest population growth was at the 12 h exposure. In parallel with the life table results, a lower population growth was obtained in the 12 and 24 h exposure in the caged prey trial than that in the caged predator trial.

## 4. Discussion

Although survival/mortality estimates and life-history traits have become the main subject in many NCEs studies, there is a need for more accurate assessments. An analysis based on a robust life table method could provide the most comprehensive information to obtain a complete understanding of predation risk on the population of prey. The developmental rate, reproduction, survival, and consumption/predation capacity of arthropods may vary depending on their biological stage and sex. Therefore, considering the stage differentiation in parthenogenetic populations such as aphids is essential for a robust life table analysis and to accurately evaluate the overall fitness of a population [34]. The age-stage, two-sex life table can accurately describe the stage differentiation while incorporating the variable rate of development that occurs among individuals. Moreover, the age-stage, two-sex life table provides an accurate relationship between the mean fecundity (*F*) and net reproductive rate (*R*_0_), which is always *R*_0_ = *F* (*N_f_*/*N*); where *N* is the initial number of nymphs used for the study and *N_f_* is the number of female adults emerged [33]. Providing the mentioned relationship is a good indication of the reliability of the results obtained by the life table analysis. 

Our results indicate that exposure to different predation risks affected the developmental time, survival rate, fecundity, and consequently, the population parameters of *S. miscanthi*. The population parameters obtained with life table analysis, in other words, the intrinsic rate of increase (*r*), finite rate of increase (λ), net reproductive rate (*R*_0_), and mean generation time (*T*) are appropriate indicators to evaluate the effect of different predation risks on biological traits (i.e., the development, survival, and fecundity) of the pest cohorts used in this study [46,53]. Compared to the control (0 h exposure) treatment, the lower *R*_0_ at 12 h exposure and shorter *T* duration at 24 h exposure in the caged predator treatment were due to differences in the preadult developmental time, survival rate, and fecundity, although the differences between the treatments in terms of survival and reproduction was insignificant. However, the lower *r*, λ, and *R*_0_, and longer *T* in the caged prey treatment for the duration of 12 and 24 h exposure were primarily due to the longer preadult developmental time and lower survival rate and reproduction. This indicates that the caged prey treatment leads to a decrease in the fitness of the pest population because even a minor decrease in the mentioned population parameters can cause a significant change in an insect population, as stated by Tuan et al. [44], Goundoudaki et al. [53], Bussaman et al. [54], Qayyum et al. [55], Zhang et al. [56], and Satishchandra et al. [57]. The population projection based on the age-stage, two-sex life table displayed a similar trend with the population parameters. As shown in our study, computer simulations based on the age-stage, two-sex life table can potentially be an invaluable tool for the evaluation of NCEs.

Our results indicated that predation risks strongly affected the development of winged polyphenism. A higher percentage of nymphs developed to alate adults under both the caged predator and caged prey treatments for 24 h exposure, which confirmed that alate polyphenism is an important biological indicator of the NCEs in *S. miscanthi*. Our findings are consistent with a previous study that proved that predation risk could induce winged adults in the cotton aphid, *Aphis gossypii* Glover (Hemiptera: Aphididae). These results suggest that the aphids showed a ‘fight or flight’ strategy by inducing winged morphs under predator risk, which could be a beneficial adaptation, facilitating aphid spread to new spaces to reduce the risk [22].

Wilson and Leather [11] reported that *S. miscanthi* shifted to nutritionally inferior resource/avoided preferred host under the *Harmonia axyridis* (Pallas) (Coleoptera: Coccinellidae) risk. Although no other studies have examined the NCEs of *C. septempunctata* on the population parameters of *S. miscanthi*, our results confirmed the results of other reports on predatory natural enemies [58,59,60]. These results suggest that the aphids also showed a ‘sit and wait’ strategy of lower population parameters [22]. Unlike that obtained in this study, exposure to the predator risk of caged *H. axyridis* over multiple generations in an agricultural system resulted in the increase in weight, development, and fecundity of *Helicoverpa armigera* (Hübner) (Lepidoptera: Noctuidae); however, it caused a reduction in its survival [17]. These results suggest that the NCEs are based on predator, prey species, and risk type. The detailed reasons for this observation need to be further examined. 

Most studies that have considered the mechanism of NCEs have focused on chemical cues, however, other mechanisms including visual or tactile cues are not well documented [61,62]. In general, insect predators use chemical cues to locate prey [63], and more recently, it has been shown that prey can eavesdrop on predator chemical cues [64]. Using an experimental arena where predators had previously foraged and were subsequently removed, researchers have isolated specific contact chemical cues as a source of information for prey. For example, one previous study suggested that fewer bird cherry-oat aphids, *R. padi*, colonize plants where the ladybeetle predators, *C. septempunctata*, had previously foraged [21]. In this study, we speculated that the prey aphids were not able to avoid predator chemical cues under the caged prey treatment, while they had more space to avoid predator chemical cues under the caged predator treatment. Therefore, the negative effect of caged prey on *S. miscanthi* is greater than on caged predator. When a prey detects the presence of a predator, the perceived threat causes fear signals and certain adaptive changes in the prey. Walking away from the threatened feeding site is one of the prominent behavioral changes observed in aphids under predation risk [22]. The caged *S. miscanthi* may have been stressed because it could not do this, which may have affected its development, reproduction, and the percentage of winged morphs. However, in this study, only female individuals of *C. septempunctata* were used for the experiment, so the potential role of the absence of males and its effects on the NCEs of female predators needs further study.

As reported in some studies, the NCEs and their associated fitness costs have the ability or potential to restrict development and reproduction, and consequently, the population growth of prey populations [65]. The NCEs may also have an impact on irreversible life-history changes over time such as changes in the reproduction or metamorphosis timing, the quality and number of progenies produced, and even the development of winged morphs [66,67]. 

Large numbers of predators are necessary to generate a significant consumptive effect on a target population. However, fewer predators may be adequate to control pest populations in the case that the NCEs have a sufficient impact on the population, as stated by Carpenter et al. [68]. Although there has been progress in understanding the role of NCEs in biological control, the information on this subject is very limited. Therefore, a large amount of research is needed to clarify and exploit the effect of the NCEs in biological control studies, thus measuring the effect of natural enemies in biological control in all aspects. Moreover, prey species may respond to the presence of predators with rapid, non-genetic phenotype changes, however, the effects of this response on the biological control outcomes have been little considered [17]. Additionally, an assortment of additional factors needs to be included when projecting predator–prey interactions (e.g., the preference of the prey stage, the randomness of predation, etc.) [42].

## 5. Conclusions

In summary, our data revealed that *S. miscanthi* could implement a complex strategy of phenotypic changes in response to predation risk. Under caged predator treatment, phenotypic changes of *S. miscanthi* included accelerated nymphal development or decelerated net reproduction rate. However, under caged prey treatment, predation risk reduced *S. miscanthi* fitness. Furthermore, *S. miscanthi* might also change to winged morphs for dispersal under both of the above treatments. These results are expected to be used in practical applications in managing this economically important pest. However, phenotypic modifications are cross-generational and appear to be mediated by the parental effects, which amplify the adaptive responses over successive generations, and may also mitigate the associated phenotypic costs. Thus, further additional studies are required to determine the broader implications, especially regarding the information on the effects of the NCEs on the biological control outcomes.

## Figures and Tables

**Table 1:** The population parameters, their definitions, and the equations used in their calculations.

Parameter and Equation	Definition
**Adult pre-reproductive period:** $\;APRP=\left(\sum_{i=1}^{N_{f}}D_{i}\right)/N_{f}$	The mean duration from the emergence of a female adult to its first reproduction.
**Total pre-reproductive period:** $\;TPRP=\left(\sum_{i=1}^{N_{f}}T_{i}\right)/N_{f}$	The mean duration from the birth of a female individual to its first reproduction. A short TPRP denotes that the cohort can produce offspring earlier.
**Age-stage survival rate:** $s_{xj}=n_{xj}/n_{01}$	The notation *s_xj_* represents the probability that a newly born individual will survive to age *x* and stage *j*, where *n*_01_ is the number of individuals used at the beginning of the life table study (i.e., the cohort size, and *n_xj_* is the number of surviving individuals at age *x* and stage *j*). The curves of *s_xj_* reveal the stage differentiation and the emergence of adults [38].
**Age-stage specific fecundity: *f_xj_***	The mean number of offspring produced by female adults at age *x*. The values *f_xj_* occurred at younger ages make a greater contribution to the intrinsic rate of increase and the finite rate of increase [32].
**Age-specific survival rate:** $l_{x}=\sum_{j=1}^{m}s_{xj}$	The probability that a newborn offspring will survive to age *x* (*m* is the number of stages). It is the pooled survival rate of all stages; therefore, it is the simplified version of *s_xj_* ignoring the stage differentiation [32].
**Age-specific fecundity:** $m_{x}=\sum_{j=1}^{m}s_{xj}f_{xj}/\sum_{j=1}^{m}s_{xj}$	The mean number of offspring produced by all surviving individuals at age *x.* If only a few individuals among all surviving ones can produce offspring at age *x*, there will be a significant gap between *f_xj_* and *m_x_* [32].
**Net reproductive rate:** $R_{0}=\sum_{x=0}^{\infty }l_{x}m_{x}$	The total number of offspring that an average individual (including females, and those died in the immature stage) can produce during its lifetime. Because *R*_0_ takes into consideration of the survival rate, it is usually less than the mean fecundity calculated based on female adults (i.e., *R*_0_ < *F*) [32].
**Intrinsic rate of increase:** $\sum_{x=0}^{\infty }\left(e^{-r\left(x+1\right)}\sum_{j=1}^{m}s_{xj}f_{xj}\right)=\sum_{x=0}^{\infty }e^{-r\left(x+1\right)}l_{x}m_{x}=1$	The population growth rate as the time approaches infinity and the population reaches the stable age-stage distribution (SASD). The population size will increase at the rate of *e^r^* per time unit. It is calculated by using the Euler–Lotka equation with age indexed from 0. The intrinsic rate is commonly used as an indicator of population fitness [32].
**The finite rate:** $\lambda =e^{r}$	The population growth rate as the time approaches infinity and the population reaches the stable age-stage distribution. The population size will increase at the rate of *λ* per time unit. The finite rate of increase is also used as an indicator of population fitness [32].
**Mean generation time:** $T=\ln R_{0}/r$	It is the time length that a population requires to increase to *R*_0_-fold of its size as the population growth rate reaches *r* and λ [32].
**Age-stage life expectancy:** $e_{xj}=\sum_{i=x}^{\infty }\sum_{y=j}^{m}s\prime_{iy}$	The time that an individual of age *x* and stage *j* is expected to live. The notation ${s^{\prime }}_{iy}$ is the probability that an individual of age *x* and stage *j* will survive to age *i* and stage *y.* It is calculated by assuming *s_xj_* = 1 [39]. Because the calculation of *e_xj_* is not based on the assumption of SASD, it can be used to predict the longevity of individuals at age *x* and stage *j*.
**Age-stage reproductive value:** $v_{xj}=\frac{e^{r(x+1)}}{s_{xj}}\sum_{i=x}^{\infty }e^{-r\left(x+1\right)}\sum_{y=j}^{m}S\prime_{iy}f_{iy}$	The contribution of an individual of age *x* and stage *j* to the future population. Individuals survived to reproductive age (i.e., *f_xj_* > 0) usually have greater *v_xj_* (i.e., greater contribution) [40,41,42,43,44].

**Table 2:** The effects of the predation risks on the developmental duration (N1 to adult) and preadult survival rate of *Sitobion miscanthi*. Values are presented by the mean ± SE.

Types of Predation Risk	Exposed Time (h)	N1 (d)	N2 (d)	N3 (d)	N4 (d)	Preadult (d)	Preadult Survival Rate
Caged predator	0	2.89 ± 0.12 Aa	2.11 ± 0.12 Aa	2.15 ± 0.12 Aa	2.26 ± 0.10 Aa	9.37 ± 0.11 Aa	0.90 ± 0.05 Aa
	12	2.22 ± 0.16 Bb	2.32 ± 0.15 Aa	2.39 ± 0.20 Aa	2.26 ± 0.14 Aa	9.09 ± 0.19 Bab	0.77 ± 0.08 Aa
	24	2.21 ± 0.14 Bb	2.23 ± 0.13 Aa	2.19 ± 0.11 Aa	2.19 ± 0.12 Ba	8.85 ± 0.23 Bb	0.87 ± 0.06 Aa
Caged prey	0	2.31 ± 0.15 Bb	2.17 ± 0.09 Aa	2.04 ± 0.06 Ab	2.39 ± 0.11 Aa	8.96 ± 0.18 Ab	0.93 ± 0.05 Aa
	12	2.88 ± 0.18 Aa	2.33 ± 0.13 Aa	2.22 ± 0.13 Aab	2.68 ± 0.19 Aa	10.05 ± 0.30 Aa	0.73 ± 0.08 Ab
	24	2.67 ± 0.15 Aab	2.35 ± 0.11 Aa	2.46 ± 0.17 Aa	2.54 ± 0.12 Aa	9.79 ± 0.25 Aa	0.80 ± 0.07 Aab

Different upper-case letters following the means within a column denote the significant differences between the caged predator and caged prey conditions at the same exposure time, whereas different lower-case letters following the means denote the significant differences among the exposure times at the same risk type based on the paired bootstrap test at a 5% significance level.

**Table 3:** The effects of the predation risks on the adult pre-reproductive period (APRP), total pre-reproductive period (TPRP), longevity, and fecundity of *Sitobion miscanthi*. Values are presented by the mean ± SE.

Types of Predation Risk	Exposure Time (h)	APRP (d)	TPRP (d)	Adult Longevity (d)	Total Longevity (d)	Reproductive Days (d)	Fecundity	Winged Morphs to the Adults
Caged predator	0	1.12 ± 0.14 Aa	10.42 ± 0.18 Aa	17.26 ± 1.03 Aa	24.43 ± 1.52 Aa	14.00 ± 0.71 Aa	43.37 ± 3.05 Aa	0.037 ± 0.037 Ab
	12	1.17 ± 0.12 Aa	10.26 ± 0.25 Ba	16.17 ± 0.95 Aa	20.33 ± 1.81 Aa	12.65 ± 0.85 Aa	37.65 ± 3.11 Aa	0.130 ± 0.071 Ab
	24	1.08 ± 0.14 Aa	9.88 ± 0.26 Ba	16.12 ± 1.18 Aa	21.97 ± 1.70 Aa	13.24 ± 0.82 Aa	39.46 ± 3.15 Aa	0.269 ± 0.089 Aa
Caged prey	0	1.15 ± 0.13 Aa	10.07 ± 0.27 Ab	14.86 ± 1.36 Aa	22.43 ± 1.60 Aa	11.33 ± 1.03 Ba	29.93 ± 2.80 Ba	0.071 ± 0.049 Ab
	12	1.05 ± 0.14 Aa	11.09 ± 0.29 Aa	13.82 ± 1.79 Aa	18.60 ± 2.09 Aa	9.86 ± 1.40 Aa	24.45 ± 3.73 Ba	0.136 ± 0.075 Ab
	24	1.27 ± 0.12 Aa	10.95 ± 0.28 Aa	12.21 ± 1.08 Ba	18.87 ± 1.50 Aa	9.73 ± 0.86 Ba	22.67 ± 2.60 Ba	0.250 ± 0.090 Aa

Different upper-case letters following the means within a column denote the significant differences between the caged predator and caged prey conditions at the same exposure time, whereas the different lower-case letters following the means denote the significant differences among the exposure times at the same risk type based on the paired bootstrap test at the 5% significance level.

**Table 4:** The effects of the predation risks on the population parameters of *Sitobion miscanthi*. Values are mean ± SE.

Types of Predation Risk	Exposed Time (h)	*r* ^†^ (d^−1^)	Λ ^‡^ (d^−1^)	*R*_0_^§^ (offspring)	*T* ^¶^ (d)
Caged predator	0	0.2294 ± 0.0066 Aa	1.2578 ± 0.0083 Aa	39.04 ± 3.57 Aa	15.96 ± 0.18 Aa
	12	0.2189 ± 0.0103 Aa	1.2447 ± 0.0128 Aa	28.87 ± 3.73 Ab	15.33 ± 0.28 Aab
	24	0.2335 ± 0.0083 Aa	1.2630 ± 0.0104 Aa	34.21 ± 3.62 Aab	15.11 ± 0.22 Ab
Caged prey	0	0.2268 ± 0.0086 Aa	1.2546 ± 0.0107 Aa	27.94 ± 2.90 Ba	14.67 ± 0.35 Bb
	12	0.1793 ± 0.0111 Bb	1.1963 ± 0.0133 Bb	17.94 ± 3.32 Bb	16.01 ± 0.45 Aa
	24	0.1878 ± 0.0109 Bb	1.2067 ± 0.0131 Bb	18.15 ± 2.61 Bb	15.39 ± 0.37 Aab

Different upper-case letters following the means within a column denote the significant differences between the caged predator and caged prey conditions at the same exposure time, whereas different lower-case letters following the means denote the significant differences among the exposure times at the same risk type based on the paired bootstrap test at the 5% significance level. ^†^ the intrinsic rate of increase, ^‡^ finite rate of increase, ^§^ net reproductive rate, ^¶^ mean generation time.

## Data Availability

The data presented in this study are available on request from the corresponding author.
